# Uncovering the burden of intentional injuries among children and adolescents in the emergency department

**DOI:** 10.1186/1471-227X-15-S2-S6

**Published:** 2015-12-11

**Authors:** Uzma Rahim Khan, Butool Hisam, Nukhba Zia, Muhammad Umer Mir, Olakunle Alonge, Seemin Jamali, Adnan A Hyder, Junaid Abdul Razzak

**Affiliations:** 1 Department of Emergency Medicine, Aga Khan Hospital, Karachi, Pakistan; 2 Johns Hopkins International Injury Research Unit, Department of International Health, Johns Hopkins Bloomberg School of Public Health, Baltimore, Maryland, USA; 3 Jinnah Postgraduate Medical Centre, Karachi, Pakistan; 4 Department of Emergency Medicine, Johns Hopkins School of Medicine, Baltimore, Maryland, USA; 5 The author was affiliated with the Department of Emergency Medicine, Aga Khan University, Karachi, Pakistan at the time when study was conducted

**Keywords:** children, adolescent, intentional injuries, Pakistan, developing countries

## Abstract

**Introduction:**

In low- and middle-income countries, injuries are a leading cause of mortality in children. Much work has been done in the context of unintentional injuries but there is limited knowledge about intentional injuries among children. The objective of this paper was to understand the characteristics of children with intentional injuries presenting to emergency departments in Pakistan.

**Methods:**

The data was from the Pakistan National Emergency Departments Surveillance (Pak-NEDS), conducted from November 2010 to March 2011 in seven major emergency departments of Pakistan. Data on 30,937 children under 18 years of age was collected. This paper reports frequency of intentional injuries and compares patient demographics, nature of injury, and discharge outcome for two categories of intentional injuries: assault and self-inflicted injuries.

**Results:**

Intentional injuries presenting to the emergency departments (EDs) accounted for 8.2% (2551/30,937) amongst all other causes for under 18 years. The boy to girl ratio was 1:0.35. Intentional injuries included assault (n = 1679, 65.8%) and self-inflicted injuries (n = 872, 34.2%). Soft tissue injuries were most commonly seen in assault injuries in boys and girls but fractures were more common in self-inflicted injuries in both genders.

**Conclusion:**

Intentional injury is one of the reasons for seeking emergency treatment amongst children and a contributor to morbidity in EDs of Pakistan. Moreover, such injuries may be underestimated due to lack of reporting and investigative resources. Early identification may be the first step leading to prevention.

## Background

Intentional injuries among children are major public health concerns, and are related to poverty, poor legal protections, illiteracy, large family size, and unemployment [1]. The 2010 Global Burden of Disease study reports that self-inflicted intentional injuries have risen by 23.8% between 1990 and 2010 in all ages [2].

Several studies in Pakistan have documented the incidence and etiology of unintentional injuries among children using surveillance data [3-9], but no study was found that has yet described the incidence, features, or outcomes of intentional injuries among children. While intentional injuries are generally underreported in most settings, the Emergency Department remains one of the first points of contact and the most important point for detection and intervention of intentional injuries, especially for severe injuries. The child's fear of the perpetrator and patriarchal notions, especially in cases of rape or sexual assault, are some of the major contributing factors to the underreporting of such events.

The purpose of this study is to determine the characteristics of intentional injuries in children and adolescents presenting at Emergency Departments in Pakistan and to document the causes and outcomes of these intentional injuries.

## Methodology

### Study design and setting

This study analyzes data from the Pakistan National Emergency Department Surveillance Study (Pak-NEDS), a pilot active surveillance of patients presenting to emergency departments (ED) of seven tertiary care hospitals purposively selected from the four provinces of Pakistan. The surveillance was conducted between November 2010 and March 2011 in: two hospitals in Karachi (capital city of Sindh province); one each in the cities of Lahore (capital city of Punjab province) and Rawalpindi (also in Punjab province); Peshawar (capital city of Khyber Pakhtunkhua province); Quetta (capital city of Baluchistan province); and Islamabad (the national capital). Two of these hospitals are private teaching hospitals, while the other five are public teaching hospitals. Approval for the study was granted by the Aga Khan University (AKU) Ethics Review Committee and the Ethics Review Boards of all participating hospitals.

### Data collection tool

A one-page standardized tool was developed based on the ambulatory care survey tool of the Centers for Disease Control and Prevention, USA and previous surveillance work done in Pakistan [10,11]. It enabled documentation of patient characteristics (age, gender, mode of arrival, presenting complaint, and discharge), injury characteristics (intent, cause, nature), and hospital characteristics (public versus private). A case was defined as anyone under 18 years with an intentional injury (including self-inflicted or assault) as reported by the patient or next of kin or medical records.

### Data collection team

Data collectors were specifically trained for this study and applied the surveillance tool to patient/next of kin interviews and to a review of medical records. The data collectors worked in three shifts to provide 24-hour coverage over the entire data collection period.

### Data Analysis

Data was entered using EpiInfo version 3.3.2 and analysis was done using SPSS version 20 [12]. Descriptive analysis of patient, injury, and hospital characteristics was performed and percentages were reported. Intentional injuries were grouped into "self-inflicted" and "assault", depending on the motive. Age was recorded as a continuous variable in the study but was later categorized into age groups to describe the relationship of injury with a specific age group in our analysis. Age was categorized into 4 groups: less than 5 years, 5-9 years, 10-14 years, and 15-17 years of age. Comparison was made between known and unknown intent as well as between self-inflicted and assault using Chi square test with p-value of <0.05. Comparison between known and unknown intent was done to determine if there was any difference in the patient characteristics of these two groups. Assault and self-inflicted injuries were also compared among males and females.

## Results

A total of 30,937 pediatric patients (under 18 years of age) were captured in the surveillance, of which 41.8% (n = 12,931) presented with injuries. The intent of injury was known for 69.4% of children (n = 8,978/12,931); of these, 28.4% were intentional injuries. The percentage of intentional injuries for children under 18 years presenting to the ED was 8.2% (2,551/30,937) of all causes.

On comparing known versus unknown intent injuries, factors such as age, gender, mode of arrival, hospital type, nature of injury, body part involved, and ED discharge information were significantly different. The percentage of girls (40.8%, 95% CIs 0.39 - 0.42), older children (47.7%, 95% CIs 0.46 - 0.49), and upper limb injuries (52.5%, 95% CIs 0.50 - 0.55) in injuries with unknown intent was significantly higher (Table 1).

**Table 1:** Comparative profile of children under 18 years of age with known and unknown intent of injury (n = 12,931)

Patient demographics	Intent known (n = 8978)	Intent unknown (n = 3953)	p-value
	
	%(95% CIs)*	%(95% CIs)*	
**Gender (n = 12,779)**			
Boys	72.3(71.4, 73.3)	59.2(58, 61)	<0.001
Girls	27.7(27, 29)	40.8(39, 42)	

**Age groups (n = 12,931)**			
Less than 5 years	17.9(17, 19)	9.5(8, 10)	<0.001
5 - 9 years	19.8(19, 21)	14(13, 15)	
10 - 14 years	32.8(32, 34)	28.8(27, 30)	
15 - l7 years	29.5(28, 30)	47.7(46, 49)	

**Hospital type (n = 12,931)**			
Public	94.8(94, 95)	92.6(92, 93)	<0.001
Private	5.2(5, 6)	7.4(7, 8)	

**Mode of arrival (n = 12,170)**			
Ambulance	6(5, 6)	3.8(3, 4)	<0.001
Non-ambulance	94.0 (93.5, 94.5)	96.1 (95.5, 96.7)	

**Nature of injury (n = 8427)**			
Soft tissue injuries	68.0(66.9, 69.1)	69.5(67.0, 71.8)	<0.001
Fractures/dislocation	19.4(18.5, 20.4)	26.8(24.5, 29.2)	
Other injuries**	12.5(11.8, 13.3)	3.8(3.0, 5.0)	

**Body parts involved (n = 10,978)**			
Head and neck	35(34, 36)	30.4(29, 32)	<0.001
Chest and abdomen	4.6(4, 5)	6.8(6, 8)	
Upper limbs	26.1(25, 27)	42.8(41, 45)	
Lower limbs	32.2(31, 33)	19.9(18, 21)	
Others	2.1(2, 2.4)	0(0, 0.2)	

**Disposition (n = 10,612)**			
ED discharge	85.6 (84.8, 86.4)	74.8 (73.2, 76.3)	<0.001
Admitted	12.6 (11.8, 13.4)	24.5 (23.0, 26.0)	
LAMA/LWBS	0.5 (0.36, 0.7)	0.2 (1.2, 5.1)	
Death	1.3 (1.1, 1.6)	0.5 (2.7, 7.8)	

*95% CIs for percentage

**Other injuries includes burns, intracranial bleed, concussion, contusion

LAMA - Left against medical advice, LWBS - Left without being seen

The majority of the children with intentional injuries were boys (74.3%). Of the total intentional injuries, 65.8% were classified as assault and the remainder as self-inflicted injuries (Table 2).

**Table 2:** Comparison of characteristics of assaults and self-inflicted injuries in children under 18 years of age (n = 2551)

Patient demographics	Total (n = 2551)	Assault (n = 1679)	Self-inflicted injuries (n = 872)	p-value**
	
	%(95% CIs)*	%(95% CIs)*	%(95% CIs)*	
**Gender (n = 2520)**				
Boys	74.3(73, 76)	77.3(75, 79)	68.6(65, 72)	<0.001
Girls	25.7(24, 27)	22.7(21, 25)	31.4(28, 35)	

**Age groups (n = 2551)**				
Less than 5 years	17.9(16, 19)	16.2(14, 18)	21.2(19, 24)	<0.001
5 - 9 years	21.4(20, 23)	19.4(18, 21)	25.2(22, 28)	
10 - 14 years	30.8(29, 33)	31.9(30, 34)	28.8(26, 32)	
15 - 17 years	29.9(28, 32)	32.6(30, 35)	24.8(22, 28)	

**Hospital type (n = 2551)**				
Public	99.2(99, 100)	99.3(99, 100)	99.1(98, 99)	0.582
Private	0.8(0.4,1)	0.7(0.3, 1)	0.9(0.4, 2)	

**Mode of arrival (n = 2369)**				
Ambulance	7(6, 8)	8.4(7, 10)	4.1(3, 6)	<0.001
Non-ambulance	93(92, 94)	91.6(90, 93)	95.9(94, 97)	

**Nature of injury (n = 1994)**				
Soft tissue injuries	53.5(51.2, 55.7)	60.1(57.4, 62.7)	39.6(35.8, 43.5)	<0.001
Fractures/dislocation	34.1(32.1, 36.2)	27.1(24.8, 29.6)	48.8(44.8, 52.7)	
Other injuries****	12.4(11.0, 14.0)	12.8(11.1, 14.7)	11.6(9.3, 14.4)	

**Body parts involved (n = 2087)**				
Head and neck	33.4(31, 35)	33.8(31, 36)	32.4(0.29, 0.36)	
Chest and abdomen	4.6(4, 6)	4.3(3, 5)	5.2(0.04, 0.07)	<0.001
Upper limbs	28.6(27, 30)	28.9(27, 31)	27.7(0.24, 0.31)	
Lower limbs	31.3(29, 33)	32.2(30, 35)	29.3(0.26, 0.33)	
Others	2.2(2, 3)	0.8(0.4, 1)	5.5(0.04, 0.07)	

**Disposition (n = 1915)**				
ED discharge	80.3 (78, 82)	80 (77, 83)	80.5 (78, 83)	0.989
Admitted	17.8 (16, 19.5)	17.9 (15.1, 21)	17.7 (15.6, 19.9)	
LAMA/LWBS	0.3 (0.001, 0.006)	0.3 (0.0005, 0.012)	0.2 (0.001, 0.008)	
Death	1.7 (1.2, 2.4)	1.8 (0.9, 3.1)	1.6 (1.02, 2.5)	

*95% CIs for percentage

** p-value for comparison between assault and self-inflicted injuries

***Other injuries includes burns, intracranial bleed, concussion, contusion

**Figure 1:**
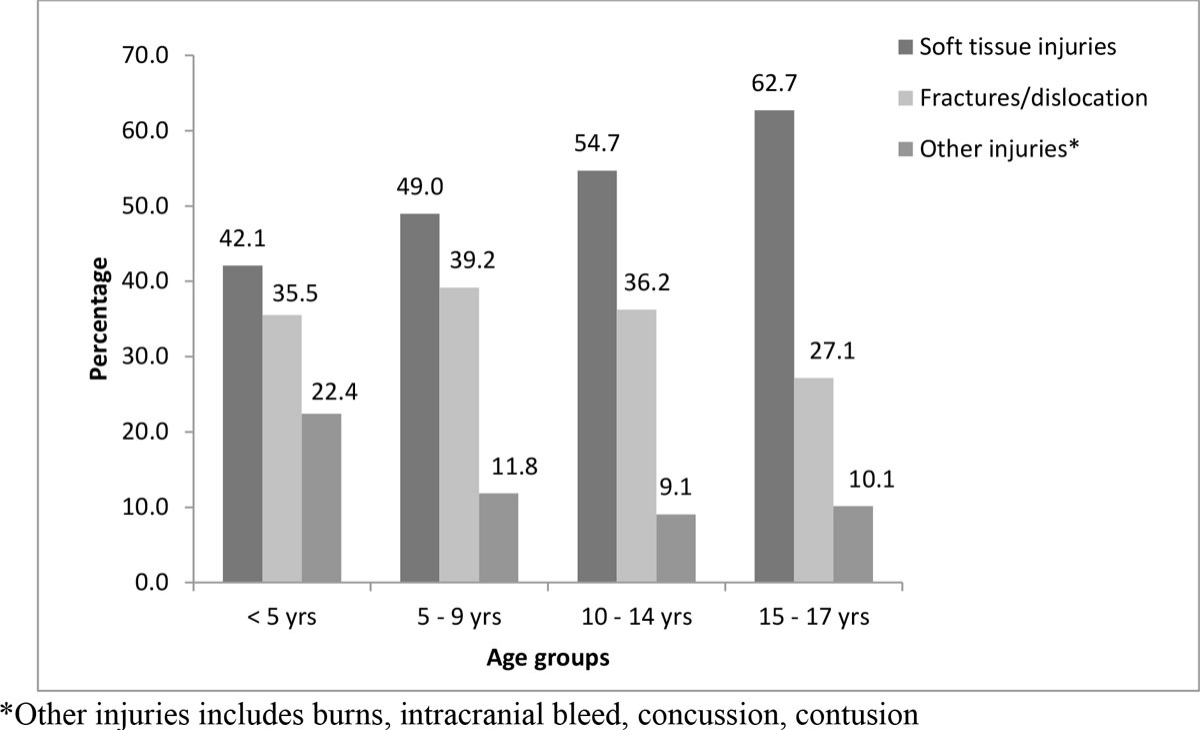
**Age wise distribution of nature of intentional injuries (n = 1994)**.

Figure 1 shows soft tissue injuries dominated throughout all age groups with an increasing trend from younger to older age groups. Fractures and dislocation showed an almost consistent pattern except a dip in the oldest age group of 15-17 years.

The boy-to-girl ratios were 1:0.29 in assault injuries and 1:0.46 in self-inflicted injuries. Among boys, the highest number of assaults and self-inflicted injuries occurred in the oldest age group (15-17 years). Among girls, self-inflicted injuries were most common in the 15-17 years age group, but assaults were more common in the 10-14 years old age group. Soft tissue injuries were most commonly seen in assault injuries in both boys and girls but fractures were more common in self-inflicted injuries in both genders. For boys, admission to the hospital was at a rate of 15% each for assault and self-inflicted injuries, compared to 22% and 24% for assault and self-inflicted injuries respectively among girls (Table 3).

**Table 3:** Gender based comparison of assault and self-inflicted injuries among children under 18 years of age (n = 2520)

Patient demographics	Assault (n = 1658) %(95% CIs)*		Self-inflicted injuries(n = 862) %(95% CIs)*	
	
	Boys (n = 1281)	Girls (n = 377)	Boys (n = 591)	Girls (n = 271)
**Age groups (n = 2520)**				
Less than 5 years	14.2(12.4, 16.3)	22.3(18.3, 26.9)	18.4(15.4, 21.9)	26.6(21.5, 32.3)
5 - 9 years	19.0(16.9, 21.3)	21.0(17.0, 25.5)	24.7(21.3, 28.4)	26.6(21.5, 32.3)
10 - 14 years	32.2(30.0, 34.9)	30.0(25.4, 34.9)	34.3(30.6, 38.4)	17.3(13.1, 22.5)
15 - 17 years	34.6(32.0, 37.3)	26.8(22.5, 31.6)	22.5(19.2, 26.1)	29.5(24.2, 35.4)

**Hospital type (n = 2520)**				
Public	99.5(98.9, 99.8)	98.7(96.8, 99.5)	99.0(97.7, 99.6)	99.6(97.6, 99.9)
Private	0.5(0.2, 1.1)	1.3(0.5, 3.3)	1.0(0.4, 2.3)	0.4(0.02, 2.4)

**Mode of arrival (n = 2343)**				
Ambulance	8.3(6.8, 10.0)	8.6(6.0, 12.2)	3.7(2.3, 5.8)	5.2(2.9, 9.0)
Public/private transport	91.7(90.0, 93.2)	91.3(87.8, 94.0)	96.3(94.2, 97.7)	94.8(91.0, 97.1)

**Nature of injury (n = 1969)**				
Soft tissue injuries	62.3(59.3, 65.3)	51.9(46.0, 57.8)	42.9(38.3, 47.6)	31.3(24.8, 38.7)
Fractures/dislocation	25.3(22.7, 28.1)	33.6(28.2, 39.4)	47.9(43.3, 52.6)	51.1(43.6, 58.8)
Other injuries****	12.4(10.5, 14.6)	14.5(10.8, 19.3)	9.2(6.8, 12.4)	17.6(12.5, 24.1)

**Body parts involved (n = 2065)**				
Head and neck	33.9(31.2, 36.8)	33.1(28.0, 38.7)	33.0(28.8, 37.6)	30.6(24.0, 37.9)
Chest and abdomen	4.5(3.4, 6.0)	3.6(1.9, 6.5)	5.1(3.3, 7.6)	5.6(2.9, 10.3)
Upper limbs	28.2(25.6, 31.0)	30.8(25.8, 36.4)	28.9(24.8, 33.3)	24.4(18.5, 31.5)
Lower limbs	32.8(30.0, 35.6)	30.8(25.8, 36.4)	29.7(25.6, 34.2)	28.3(22, 35.6)
Others	0.5(0.2, 1.2)	1.6(0.6, 4.0)	3.3(2.0, 5.5)	11.1(7.1, 16.9)

**Disposition (n = 1870)**				
ED discharge	82.9(80.3, 85.3)	75.3(69.9, 80.1)	83.2(79.4, 86.5)	74.2(67.7, 79.8)
Admitted	15.5(13.3, 18.1)	21.9(17.4, 27.2)	14.6(11.5, 18.2)	24.0(18.6, 30.3)
LAMA/LWBS	0.2(0.04, 0.9)	0	0.2(0.01, 1.4)	0.5(0.02, 2.9)
Death	1.3(0.7, 2.4)	2.7(1.3, 5.5)	2.0(1.0, 3.9)	1.4(0.4, 4.3)

*95% CIs for percentage

** p-value for comparison between assault and self-inflicted injuries

***Other injuries includes burns, intracranial bleed, concussion, contusion

## Discussion

The burden of intentional injuries accounted for 8.2% of all children visiting EDs and 19.7% of all injuries in this study. Earlier statistics vary depending on the methodology or setting. A self-reported study of mothers in a private tertiary care hospital of Karachi, Pakistan showed 25.5% children between 6 to 12 years of age were physically abused by either parent during the last 12 months [13]. In southwest Nigeria, non-accidental injuries accounted for 0.84% of all 5264 patients and 21.3% of the 207 injured patients at a tertiary hospital [14]. A recent study from South Africa found that 7.4% of pediatric injuries presenting to the ED of a tertiary care hospital over a period of 10 years were intentional injuries [15]. Another retrospective cohort study from Western Australia found that only 0.03% of all ED presentations and 0.2% of all intentional injury presentations of pediatric patients over a period of 4 years were identified as maltreatment cases [16].

There are various reasons for intentional injuries in children. For example, one study found an association between county mean income and percentage of families living below the poverty line and intentional injury rate, suggesting that financial hardship may be an important risk factor for these injuries [17]. Moreover, Sethi et al summarized general risk factors that influence maltreatment/neglect/abuse, including: a tolerance for violence by society, communities, and families, particularly in the case of domestic violence; social norms that encourage or accept the corporal punishment of children; gender and social inequality; lack of or inadequate housing including living in social housing; lack of services to support families and institutions and to meet specialized needs; high levels of unemployment; poverty; and alcohol and drug abuse [18].

The study also found missed opportunities for identifying intentional injuries, as is evident from our results which show 30% of injuries with no information on intent in their medical records. Underreporting of intentional injury is already known [19]. Identification and reporting depend upon the expertise of the treating physician on the patterns of intentional injuries. Checklists such as the Quality Improvement Report by Benger & Pearce (2002) could aid healthcare providers in asserting if an injury is intentional or not [20]. Future studies could also assess the effectiveness of such checklists in improving the reporting of intentional injuries in low- and middle-income countries. Training of ED physicians and familiarizing them with the legal and social aspects of reporting child abuse may help improve detection rates. As one study found, adopting uniform screening guidelines can prove beneficial for detection and reporting of child abuse [21].

In Pakistan, protection of the child falls solely under the *Convention of The Rights of The Child *which was ratified in 1990. The law interferes in family matters only when the family breaks down, in which case the law gives preference to the next of kin or the extended family in granting responsibility for the guidance of the child. Intentional injuries, if identified by physicians, are required to be reported to the police; this sometimes leads caregivers to go against medical advice by taking the child away from the hospital. There are no child protection agencies that can act on behalf of the child using evidence from ED physicians. There are some non-governmental organizations in Pakistan such as Sahil and Konpal, which work to implement child protections, especially against child abuse [22]. A collaborative effort should be implemented between ED physicians and social organizations to coordinate all efforts in protecting the child [23].

In developed countries, there are legal implications for reporting child abuse. For example, in the United States, once reports are sent to Child Protective Services regarding any form of child abuse and found to be substantiated, the agencies can initiate court actions to protect the child.

Failure to report can lead to continuation of abuse. Such childhood adversities put children at risk for several mental health problems in the future including Major Depressive Disorder, Borderline Personality Disorder, and Schizophrenia. Moreover, these issues could deter them from playing a positive role in society.

Our results show that children presenting with injuries with intent known and those with intent unknown are different with respect to their age, gender, hospital type, and mode of arrival. For instance, the proportion of girls with unknown intent was higher compared to girls with known intent. It is reasonable to assume that a significant portion of this missing information could be related to intentional injuries that remained undetected; this could be due to caregivers not sharing the details of an intentional injury due to social desirability, or due to a lack of physicians' knowledge about related clinical factors. Mutual trust between physician and caregiver should be built without pointing fingers in order to identify such cases.

It is intriguing to note that no case of sexual assault was reported in this study. Various factors may be the cause of this. In Pakistan, sexual abuse is very much a 'taboo' topic and has consequently largely been neglected. It is perceived as something shameful and the victim is often looked down upon as a source of humiliation instead of being given the support and care that is needed. Poverty and lack of education have only worsened the issue as most families are left unaware of the rights granted to the child. With regards to the emergency physician, all the factors pointed out earlier (i.e. lack of physician training, screening guidelines, and child protection services) may have led to underreporting.

Although more boys presented compared to girls in both the categories of assaults (77% vs 23%) and self-inflicted injuries (69% vs 31%), the boy-to-girl ratio is vastly reduced for self-inflicted injuries. This is consistent with results from a previous study that found the female gender to be a risk factor for deliberate self-harm or self-inflicted injuries [24-26]. This may be explained by their increased vulnerability to depression, social roles and pressure, and cultural norms. Depression is one of the most important risk factors for suicide in young girls [27-29].

Overall, this study found that soft tissue injuries and then fractures were the most common injuries in children with intentional injuries overall as well as in the assault group. Soft tissue injuries (contusion, bruises, abrasion, and open wounds) were reported previously in assaults [17,30-32]. The fact that the majority of self-inflicted injuries resulted in fractures and dislocations, particularly in girls, is not consistent with earlier reported results which showed poisoning or soft tissue injuries were most common even in self-inflicted injuries [33,34]. This distinction is worth exploring, as the sub-analysis of the mechanism of intentional injuries (not shown in the paper) showed pushing/shoving to be the major cause of intentional injuries. However, it is also important to note that mechanism was only available for 13% of cases. The high number of fractures could also be related to other factors such as Vitamin D deficiency in the Pakistan population, especially in girls [35,36]. This finding has implications for future prevention; for instance, programs could be designed to limit the exposure of high-risk individuals to heights (using supervision and family support) [37,38]. Similarly, stricter regulations and control of penetration and sharp objects within a child's environment could be put in place to minimize assaults [39].

While our study presents a broad scope regarding the epidemiology of childhood intentional injuries, the estimates stated in our study might not be generalizable to tertiary hospitals of rural areas, because the data was only collected from hospitals of large urban cities; however, these hospitals do have some coverage of surrounding rural localities.

## Conclusion

Intentional injuries contribute a significant burden on emergency departments of Pakistan. The findings of our study have far-reaching implications. Firstly, the study describes the reality of intentional injuries, an issue that was largely neglected until recently highlighted by a few social organizations. The study presents nationwide data with specific descriptive facts relating to age, gender, type of injury, mode of arrival, and other variables. Secondly, the results of this study help highlight the need for further intentional injury research, and for the development of a protocol in emergency departments in Pakistan that identifies intentional injuries in children and assists in the counsel of these children and their families. Lastly, the methodology of this study could be applied in other low- and middle-income countries where child abuse is still a neglected issue.

## Competing interests

The authors declare that they have no competing interests.

## Authors' contributions

JR and URK conceptualized the topic for the paper. BH, URK, NZ, MUM and OA were involved in draft writing. NZ and URK conducted analysis. URK, NZ, MUM, OA, AAH and JAR critically reviewed the draft. All authors read and approved the final draft before submission.
